# Preoperative Elevated Platelet Count as a Prognostic Factor in Vulvar Squamous Cell Cancer: A Mini-review

**DOI:** 10.7759/cureus.2279

**Published:** 2018-03-06

**Authors:** Ahmed Abu-Zaid, Osama Alomar, Hany Salem

**Affiliations:** 1 College of Graduate Health Sciences, University of Tennessee Health Science Center, Memphis, Tennessee, United States; 2 Department of Obstetrics and Gynecology, King Faisal Specialist Hospital and Research Centre, Riyadh, Saudi Arabia

**Keywords:** platelet count, thrombocytosis, prognosis, survival, vulvar cancer

## Abstract

Preoperative thrombocytosis has been shown to be a marker of advanced disease and poor survival in gynecologic malignancies, specifically endometrial, ovarian, and cervical cancers. The aim of this study is to provide a focused mini-review on all the existing literature concerning the role of preoperative thrombocytosis as a prognostic factor in vulvar squamous cell cancer (SCC). A PubMed search (until February 20, 2018) of all peer-reviewed and English-published articles was conducted using the following keywords: platelet, thrombocytosis, and vulvar cancer. Only three studies met the search protocol. It is concluded that preoperative thrombocytosis does not emerge as a substantial independent prognostic factor of disease-free survival (DFS) and overall survival (OS) in patients with vulvar SCC. Nevertheless, the interpretation of this conclusion should be done with extreme cautiousness. This can be ascribed to the heterogeneity of the reported data across the three studies, especially concerns pertaining to methodological designs. Additional related uniform studies are needed, so that data can be usefully pooled into a well-characterized systematic review/meta-analysis study, in order to devise valid mathematically proven conclusions. For now, International Federation of Gynecology and Obstetrics/ Fédération Internationale de Gynécologie et d’Obstétrique staging (FIGO staging) and inguino-femoral lymph node involvement continue to be the most established independent prognostic factors of DFS and OS in patients with vulvar SCC.

## Introduction and background

Tumor-platelet interactions in solid tumors have been previously characterized [1, 2]. From a molecular cancer point of view, thrombocytosis has been illustrated to be stimulated by cancer-mediated production of cytokines, most commonly interleukins (IL-1, IL-3, IL-6, and IL-9) and granulocyte-macrophage colony-stimulating factor (GM-CSF). These produced cytokines eventually promote thrombopoiesis and megakaryocyte proliferation. In addition, cancerous cells secrete a powerful platelet-activating substance known as thrombin. As a consequence, thrombin-activated platelets produce diverse proteins that may trigger angiogenesis, particularly platelet-derived growth factor (PDGF) and vascular endothelial growth factor (VEGF). Eventually, angiogenesis will boost cancer proliferation and metastatic dissemination. From a clinical point of view, it has been demonstrated that thrombocytosis is linked to poor clinicopathological and unfavorable survival outcomes. From a therapeutic point of view, it has been determined that pharmacological platelet inhibition or reduction (for example, anti-IL-6 and heparinoids) diminishes the propensity of metastatic spread and offers survival benefits. However, these pharmacological interventions are associated with unwanted interference with normal platelet hemostasis, thus triggering bleeding-related adverse events.

Preoperative thrombocytosis has been shown to be a marker of advanced disease and poor survival in gynecologic malignancies. This is specifically true for endometrial, ovarian, and cervical cancers [3].

The aim of this mini-review is to briefly review all the existing literature concerning the role of preoperative thrombocytosis as a prognostic factor in patients with vulvar squamous cell cancer (SCC). A PubMed search of all peer-reviewed and English-published articles was conducted using the following keywords: platelet, thrombocytosis, and vulvar cancer. All articles published until February 20, 2018 were included in the mini-review.

## Review

There were only three (n=3) studies that met the search protocol [4-6]. Table 1 summarizes the key findings of all PubMed-indexed, peer-reviewed, and English-published literature on the role of preoperative thrombocytosis as a prognostic factor in patients with vulvar SCC until February 20, 2018.

**Table 1:** Summary of all PubMed-indexed, peer-reviewed, and English-published literature on the role of preoperative thrombocytosis as a prognostic factor in patients with vulvar squamous cell cancer until February 20, 2018 (n=3) DFS: disease-free survival; FU: follow-up; LN: lymph node; LVSI: lympho-vascular space invasion; n: patient sample size; OS: overall survival; Ref: reference; USA: United States of America

Ref	Authors	Country	Year	Thrombocytosis cut-off	n	Patient groups			Key findings of the study
						Group	n (%)	5-year OS rate (%)	
[4]	Lavie et al.	England	1999	≥400 x 10^9^/L	181	(+) Thrombocytosis	28 (15.5)	87.3	Mean platelet count was 313 x 10^9^/L (range: 139 x 10^9^/L – 593 x 10^9^/L) Thrombocytosis was associated with anemia (p=0.0016) and leukocytosis (p=0.0001) Thrombocytosis was not associated with FIGO stage (p=0.549), metastatic groin LNs (p=0.94) and metastatic pelvic LNs (p=0.891) The 5-year OS was not statistically different (p=0.586) Cox regression: Thrombocytosis was not a statistically significant independent prognostic factor of DFS (p=0.2); tumor histology, tumor number and FIGO stage were so (p=0.003, p=0.003 & p=0.0001, respectively) Follow-up duration: not mentioned
						(–) Thrombocytosis	153 (84.5)	76.5	
[5]	Hefler et al.	USA	2000	>300 x 10^9^/L	62	(+) Thrombocytosis	17 (27.4)	25	Median platelet count was 268.5 x 10^9^/L (range: 88 x 10^9^/L – 778 x 10^9^/L) Platelet count was statistically associated with tumor grade (p=0.01) but not age or FIGO stage Thrombocytosis was significantly associated with worse DFS (p=0.003) and OS (p<0.001) Cox regression: Thrombocytosis was not a statistically significant independent prognostic factor of DFS (p=0.2) and OS (p=0.5); FIGO stage was so for both DFS (p=0.003) and OS (p=0.04) Follow-up duration (range): 0.5–72 months
						(–) Thrombocytosis	45 (72.6)	87.5	
[6]	Uysal et al.	Turkey	2013	>450 x 10^9^/L	41	(+) Thrombocytosis	8 (19.5)	75	Mean platelet count was 335.4 x 10^9^/L (range: 142 x 10^9^/L – 1115 x 10^9^/L) Thrombocytosis was not statistically associated with LN spread (p=0.93), FIGO stage (p=0.78), tumor grade (p=0.65), LVSI (p=0.82), tumor size (p=0.73), tumor depth invasion (p=0.18) and surgical margins (p=0.31) The 5-year OS was not statistically different (p=0.75) Follow-up duration (range): 60–213 months
						(–) Thrombocytosis	33 (80.5)	66.7	

In 1999, from England (Queen Elizabeth Hospital, Gateshead), Lavie and partners [4] reported their experience of preoperative thrombocytosis as a prognostic factor in 201 patients with vulvar cancer. Preoperative thrombocytosis was defined as a platelet count ≥400 x 10^9^/L. A total of 181 patients (90%) had vulvar SCC histology, and the prevalence of preoperative thrombocytosis was 15.5% (n=28). The overall mean platelet count was 313 x 10^9^/L (range: 139 x 10^9^/L – 593 x 10^9^/L). Patients with preoperative thrombocytosis had higher statistically significant anemia (p=0.0016) and leukocytosis (p=0.0001) when compared to patients with normal preoperative platelet counts. Moreover, no statistically significant differences were found between both groups with regards to the following clinicopathological parameters: age, marital status, parity, menopause, presenting symptoms, clinically palpable groin lymph nodes, tumor appearance/size/number, metastatic inguino-femoral lymph nodes, and FIGO stage. (FIGO stands for International Federation of Gynecology and Obstetrics/ Fédération Internationale de Gynécologie et d’Obstétrique). Most importantly, the five-year overall survival (OS) rate was not statistically significantly different between both groups (p=0.586). A multivariate step-wise Cox regression analysis revealed that preoperative thrombocytosis was not a statistically significant independent prognostic factor of disease-free survival (DFS, p=0.2). Instead, tumor histology, tumor number, and FIGO stage were the only significantly independent prognostic factors of DFS (p=0.003, p=0.003, and p=0.0001, respectively). The study concluded that preoperative thrombocytosis was not a significant independent prognostic factor of survival in patients with vulvar SCC.

In 2000, from United States of America (University Hospital of Vienna, Texas), Hefler and colleagues [5] reported their experience of preoperative thrombocytosis as a prognostic factor in 62 patients with vulvar SCC. Preoperative thrombocytosis was defined as a platelet count >300 x 10^9^/L. The overall prevalence of preoperative thrombocytosis was 27.4% (n=17). The overall median platelet count was 268.5 x 10^9^/L (range: 88 x 10^9^/L – 778 x 10^9^/L), and it was significantly associated with tumor grade (p=0.01) but not age of patient and FIGO stage. In a univariate analysis, patients with preoperative thrombocytosis had statistically significant worse DFS (p=0.003) and OS (p<0.001) when compared to patients with normal preoperative platelet counts. A multivariate step-wise Cox regression analysis revealed that preoperative thrombocytosis was not a statistically significant independent prognostic factor of DFS and OS. Instead, the analysis revealed that FIGO stage was the only significantly independent prognostic factor of DFS (p=0.003) and OS (p=0.04). The study concluded that preoperative thrombocytosis was not a significant independent prognostic factor of survival in patients with vulvar SCC.

In 2013, from Turkey (Aegean Maternity and Teaching Hospital, Izmir), Uysal and associates [6] reported their experience of preoperative thrombocytosis as a prognostic factor in 41 patients with vulvar SCC. Preoperative thrombocytosis was defined as a platelet count >450 x 10^9^/L. The overall prevalence of preoperative thrombocytosis was 19.5% (n=8). The overall mean platelet count was 335.4 x 10^9^/L (range: 142 x 10^9^/L – 1115 x 10^9^/L). In a univariate analysis, no statistically significant correlations were identified between preoperative thrombocytosis and various clinicopathological prognostic parameters including tumor size/grade/depth, lympho-vascular space invasion, metastatic lymph node spread, and FIGO stage. The five-year OS did not differ statistically between patients with and without preoperative thrombocytosis (p=0.75). The study concluded that preoperative thrombocytosis was not a significant independent prognostic factor of survival in patients with vulvar SCC.

Thus, in reference to the body of existing literature [4-6], it can be deduced that preoperative thrombocytosis does not emerge as a substantial independent prognostic factor of DFS and OS in patients with vulvar SCC. Nevertheless, the interpretation of this conclusion should be done with extreme cautiousness [7]. This can be largely ascribed to the heterogeneity of reported data across studies in terms of concerns pertaining to methodological designs. The most important concern was the employment of different cut-offs in defining thrombocytosis. Moreover, the following study limitations contribute to the heterogeneity of data: (i) small-sized study samples, (ii) relatively short and unspecified follow-up intervals, (iii) absent univariate/multivariate calculations of DFS, (iv) the inconsistent staging surgery procedures (that is, presence/absence of inguino-femoral lymph node dissections), and (v) failure to describe when and how blood platelet counts were measured.

Taken together, the above-mentioned study-related shortcomings prevented us from conducting a strong evidence meta-analysis study protocol. There is, undoubtedly, a need for more uniform and strongly-designed studies on the role of preoperative thrombocytosis as a prognostic factor in vulvar SCC. Later, these data can be usefully pooled into well-characterized systematic review and meta-analysis study protocols, in order to deduce valid mathematically proven conclusions.

## Conclusions

Preoperative thrombocytosis does not emerge as a substantial independent prognostic factor of DFS and OS in patients with vulvar SCC. The validity limitation of this conclusion should be acknowledged in reference to the heterogeneity of data reported in these studies. In the meantime, FIGO staging and inguino-femoral lymph node involvement continue to be the most established independent prognostic factors of DFS and OS in patients with vulvar cancer. Lastly, there is always a continuous requisite to identify convenient (general blood parameters) and novel (clinical/pathological) prognostic markers of survival and likelihood of treatment responsiveness in patients with vulvar SCC—this is an important domain for prospective biomedical research.
